# Dr. Rudolph August Witthaus

**Published:** 1916-01

**Authors:** 


**Dr. Rudolph August Witthaus,** N. Y. University 1875, died at his home in New York City, December 22, aged 64. He was Professor of Chemistry in the Medical Dept., University of Buffalo, 1882-1889 though, for the last two years of his service, he was not a resident of Buffalo and gave only a few lectures each session. In 1898, he became Professor of Chemistry in Cornell University, Medical Dept., becoming Emeritus Professor in 1911. He was the author of several works on chemistry for use by medical students and practitioners and, conjointly with Tracy C. Becker of Buffalo, of an elaborate work on Medical Jurisprudence. He was well known as an expert witness in poisoning cases. Few men had done so much to advance the practical application of chemistry to medicine and to emphasize its importance as an undergraduate medical study, in the '80's although he was a toxicologist rather than a physiologic chemist. An anecdote of his connection with the University of Buffalo illustrates his keen wit. Though generally well liked, and always highly esteemed, and recognized as an excellent teacher, he had at one time fallen into temporary disfavor on account of participating in the movement toward more stringent examinations than were the custom 30 or 40 years ago in medical schools. Accordingly, as he entered the lecture room, he was hissed. His response was "Gentlemen, there are two animals that hiss: the snake which I crush with my heel and the goose which I pluck."

